# Editorial for Special Issue “Perspectives of Immunotherapy in Tumors of the Gastrointestinal Tract”

**DOI:** 10.3390/cancers15041223

**Published:** 2023-02-15

**Authors:** Gianluca Masi

**Affiliations:** 1 Department of Translational Research and New Technologies in Medicine and Surgery, University of Pisa, 56126 Pisa, Italy; gianluca.masi@unipi.it; 2 Division of Medical Oncology, Azienda Ospedaliero-Universitaria Pisana, 56126 Pisa, Italy

After transforming the therapeutic perspective of many solid neoplasms, immunotherapy is finally making its way in the setting of gastro-intestinal (GI) primary cancers. Both monoclonal antibodies targeting PD(L)-1 or CTLA-4 and newer strategies, such as vaccines, have started to undermine the chemotherapy-exclusive paradigm of oesophageal, gastric, liver, and colorectal carcinomas. Either in combination with cytotoxic regimens or as mono-therapies, chemotherapeutics are bringing greater efficacy and milder adverse events to different-stage GI patients. However, the broad biological heterogeneity of GI neoplasms is mirrored by an uneven benefit derived from this strategy.

With our Special Issue “Perspectives of Immunotherapy in Tumors of the Gastrointestinal Tract”, we aimed to pinpoint the most relevant milestones achieved by immunotherapy in these diseases, while pointing out the weaknesses of this approach which still need to be tackled, such as primary and secondary resistance mechanisms and predictive biomarkers of the outcome.

The first aspect underlined by Borelli et al. [1] and Salani et al. [2] in their review contributions is the existence of a non-negligible rate of primary and secondary resistance to immunotherapy, also in the setting of deficient mismatch repair (dMMR) metastatic colorectal cancer (mCRC) and advanced hepatocellular carcinoma (aHCC), respectively. In spite of a great outcome improvement demonstrated in their pivotal trials, the rate of patients not deriving any benefit from first line immunotherapy exceeds 15% in both mCRC and aHCC. Suggested strategies to overcome it are based on treatment intensification, patient selection refinement (such as through the determination of TMB, PD-L1, and POLE/D in mCRC), and the identification of alternative immunitary targets for drug development (such as IDO, LAG-3, and TIM-3 in both diseases). All these possibilities are explored.

The second aspect brought to attention by Raghad Khalid AL-Ishaq et al. [3] and by the original contribution of Jan Hrudka et al. [4] is the need for a deeper biological knowledge of the mechanisms orchestrating the immunitary response in order to tailor the application of currently in-use immunotherapy and to guide the discovery of new drugs. The first example is given by the role of gut microbiome: the contribution exhaustively summarizes the up-to-date knowledge of microbiome involvement in GI carcinogenesis, from inflammation to cellular proliferation, metastasis, and apoptosis, and recapitulates the preliminary evidence of its influence on immunotherapy efficacy. The second example is given by the prognostic and putative therapeutic significance of various heat shock proteins (HSP) in CRC cancer, in a wide retrospective cohort of 297 tissue samples. Despite being significantly more prevalent in pMMR, HSP are known to interfere with the function of T cells in response to different stressors, thus warranting their investigation as co targets with known checkpoints.

Additional evidence of the importance of the biological knowledge of immunitary determinants is provided by Shria Kumar et al. [5]. The authors reviewed the clinic–epidemiological knowledge of Lynch syndrome in upper GI cancers, focusing on the central role of secondary prevention to detect early diagnoses, thus improving the patient outcome.

Lastly, following the astonishing results obtained in the aforementioned settings, there are new developments in immunotherapy which were previously deemed refractory, pMMR locally advanced rectal cancer and pMMR mCRC, where the addition of immunotherapy to radiotherapy and/or chemotherapy backbones is showing promising results, as discussed by Germani M.M et al. [6,7].

In conclusion, the different contributions to this Special Issue address complementary aspects of the recent employment of immunotherapy in upper GI cancers, which constitutes one of the greater breakthroughs in the last five years of oncology. Through this collection, we hope we have provided various interesting subjects to guide further research in this area.
